# Predictive Value of Tumor Regression Grading on the Prognosis of Neoadjuvant Chemotherapy for Locally Advanced Gastric Cancer: A Systematic Review and Meta-Analysis

**DOI:** 10.14309/ctg.0000000000000860

**Published:** 2025-05-14

**Authors:** Ruchen Wu, Shuying Lin, Junze Chen, Gang Wang, Lulu Han

**Affiliations:** 1 First Clinical Medical College, Xuzhou Medical University, Xuzhou, Jiangsu, China;; 2 Endoscopy Room, Quanzhou First Hospital Affiliated to Fujian Medical University, Quanzhou, Fujian, China;; 3 Cancer Institute, Xuzhou Medical University, Xuzhou, Jiangsu, China.

**Keywords:** tumor regression grade, local advanced gastric cancer, neoadjuvant chemotherapy, overall survival, meta-analysis

## Abstract

**INTRODUCTION:**

Tumor regression grade (TRG) after neoadjuvant chemotherapy is recognized as a significant and favorable prognostic indicator in various cancer types. However, this relationship remains less defined and has not been systematically investigated in locally advanced gastric cancer (LAGC). To address this gap, we conducted a meta-analysis aimed at assessing the prognostic influence of tumor regression after preoperative therapy on disease-free survival (DFS) and overall survival (OS) among patients with LAGC.

**METHODS:**

A systematic search was conducted across the following databases: PubMed, Web of Science, Embase, Cochrane, WF, CNKI, SinoMed, and VIP. The primary outcomes included DFS and OS, estimated using hazard ratios (HRs) and corresponding 95% confidence intervals (CIs). Subsequently, either the fixed-effects model or the random-effects model was used to compute HR and 95% CI based on the results of heterogeneity analysis.

**RESULTS:**

A total of 11 studies, comprising 2,733 patients, were included in the final analysis. The results indicated that a lower TRG was associated with prolonged DFS (HR 0.53, 95% CI 0.32–0.88) and prolonged OS (HR 0.59, 95% CI 0.39–0.87) in patients with LAGC who received neoadjuvant chemotherapy. Sensitivity analysis demonstrated that no single study significantly influenced the results for both DFS and OS. Publication bias was identified in the meta-analysis for OS, whereas no publication bias was detected in the meta-analysis for DFS.

**DISCUSSION:**

A lower TRG score is associated with improved DFS and OS in patients with LAGC receiving neoadjuvant chemotherapy.

## Graphical abstract

**Figure:**
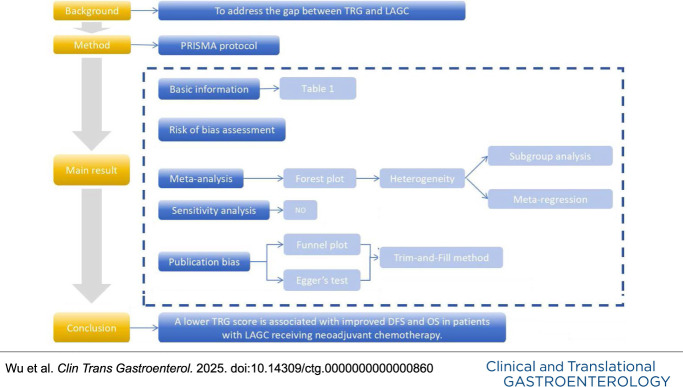

## INTRODUCTION

Gastric cancer (GC) is a prevalent malignant tumor globally (1), particularly in East Asia (2), and ranks as the third leading cause of cancer-related mortality (3). In East Asian countries such as China, Japan, and South Korea, the incidence and mortality rates of GC are significantly higher than those in other regions (2). Locally advanced gastric cancer (LAGC) is characterized by significant aggressiveness and a high risk of recurrence and metastasis. It is defined as pathologically confirmed T3/T4 or N+ (American Joint Committee on Cancer eighth edition) gastric adenocarcinoma without distant metastasis (M0), requiring neoadjuvant chemotherapy followed by curative resection. Therefore, early diagnosis and effective treatment are essential for improving patient prognosis (4).

For patients with LAGC, neoadjuvant chemotherapy has emerged as a standard treatment option (5). This approach not only reduces tumor size and stage but also enhances surgical resection rates and long-term survival (6). However, considerable variability exists in the response to neoadjuvant chemotherapy among patients. Therefore, it is essential to identify effective biomarkers for predicting treatment response and prognosis.

Tumor regression grade (TRG) is a quantitative method used to evaluate pathological changes in tumor tissue after neoadjuvant chemotherapy. It reflects a patient's response to treatment by assessing tumor remnants posttherapy (7). Numerous studies have reported the predictive value of various TRG scoring systems concerning the prognosis of patients with LAGC (8).

The morphological manifestations of gross tumor specimens were initially evaluated using Mandard criteria: (i) obvious residual tumors, including ulcerative, mushroom-like, and infiltrating types; (ii) significant tumor regression; and (iii) scarring manifestations. TRG was quantified into 5 distinct grades based on histomorphology. A clear correlation has been established between TRG and patient survival (9). Becker et al proposed a grading system for tumor regression in patients with LAGC who received cisplatin-based neoadjuvant chemotherapy. This system classifies tumor regression into 3 grades: (i) TRG 1a: no tumor cells present in the tumor bed; (ii) TRG 1b: less than 10% residual tumor; (iii) TRG 2: 10%–50% residual tumor; and (iv) TRG 3: greater than 50% residual tumor (10). The Becker system has also been applied to other gastrointestinal tumors (11,12). Other TRG systems, such as the Dworak (13) and Rödel (14) systems, are commonly used to estimate tumor regression. TRG is highly recommended for evaluating the prognosis of LAGC after neoadjuvant therapy (7). Nonetheless, the conclusions drawn from various studies are inconsistent, potentially due to differences in study design, sample size, and assessment methods.

In this study, a systematic review and meta-analysis were adopted to comprehensively evaluate the correlation of TRG with the prognosis of patients with LAGC after neoadjuvant chemotherapy. After a thorough search of relevant literature, rigorous selection of eligible studies, and an assessment of study quality, statistical methods were used to summarize the results of multiple studies, thereby improving the reliability and accuracy of the evidence.

## METHODS

This study followed the Preferred Reporting Items for Systematic reviews and Meta-Analyses statements (15). A protocol was registered at PROSPERO (CRD42024583573) before the literature search.

### Data sources

The databases searched included PubMed, Web of Science, Embase, Cochrane, WF, CNKI, SinoMed, and VIP, focusing on studies published in English and Chinese regarding the relationship between TRG and the prognosis of GC after neoadjuvant chemotherapy. Medical Subject Headings terms and free-text terms related to GC and TRG based on the PECOS principle were used as the search strategy, with Boolean logic connectors used to construct the search query (see Supplementary Table S1, Supplementary Digital Content 1, http://links.lww.com/CTG/B314).

### Study selection

Eligible studies met the following criteria: (i) patients undergoing gastrectomy for GC after neoadjuvant therapy; (ii) patients were assigned to 2 groups: those who had better conditions with TRG score and those who had poor conditions with TRG score; (iii) primary outcomes included disease-free survival (DFS) and overall survival (OS); (iv) studies provided data on hazard ratio (HR) and 95% confidence interval (CI); and (v) the types of studies were mainly case-control studies and cohort studies.

Two researchers independently scanned the titles and abstracts of articles retrieved, downloaded the full texts, and evaluated them to determine the finally included studies. Any disagreements on study selection were tackled through discussion.

### Data extraction

Two independent researchers (Wu and Lin) performed initial data extraction. A third researcher (Chen) merged the data and resolved discrepancies by reviewing the original text. Unresolved disputes were addressed through joint discussions among all 3 researchers (Wu, Lin, and Chen). For persistent disagreements, senior researchers (Wang and Han) were consulted for final arbitration. The data extracted in the article were all from the inception of the databases to August 30, 2024. Extracted data covered the first author, publication year, country, sample size, age, sex ratio, types of TRG, varieties of grouping, and HRs (95% CIs) for OS and DFS.

### Quality assessment

The quality of included cohort and case-control studies was independently appraised by 2 researchers using the Newcastle-Ottawa scale in 3 main dimensions: selection, comparability, and ascertainment of outcomes. In addition, the quality was graded into low, medium, and high levels, with stratified analysis performed as appropriate to ensure the rigor of the analysis.

### Data synthesis

Data analysis was made in Stata 15 software. The *I*^2^ statistic test was used to evaluate the heterogeneity of the pooled outcomes. A random-effects model was considered if *I*^2^ value >50% and *P* value <0.05; otherwise, a fixed-effects model was applied. The key outcome measure was HR for OS and DFS. To delve deeper into the potential sources of heterogeneity, the leave-one-out approach and stratified analysis were leveraged, for evidence grading of original studies and TRG scoring systems. Furthermore, funnel plots and Egger test were adopted to appraise potential publication bias.

## RESULTS

### Result of study selection

**Figure 1.**
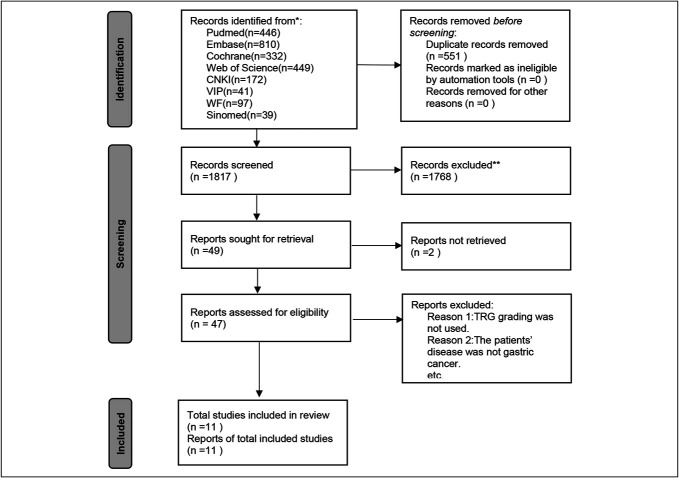
Flowchart of literature screening.

A total of 2,386 articles were retrieved from the initial search. Of these, 551 duplicate articles were excluded. A total of 1,768 articles were excluded after the review of titles and abstracts. Two articles lacked accessible full texts, and 36 articles were excluded after a thorough full-text review (Figure 1).

This meta-analysis incorporated 11 studies (16–26), encompassing a total of 2,733 patients. The 11 articles were published between 2019 and 2024. Most research was conducted in China, while the remaining studies were performed in the United States, Italy, France, and Switzerland. The Mandard scale was the most frequently used method for TRG scoring, while the other scoring systems included Becker and CAP. The main chemotherapy regimens included FOLFOX (folinic acid, fluorouracil, and oxaliplatin), SOX (S-1, oxaliplatin), XELOX (capecitabine and oxaliplatin), ECF (epirubicin, cisplatin, and fluorouracil), and ECX (epirubicin, cisplatin, capecitabine). However, immunotherapy was not included in any of the treatment protocols. Tumor size, location, histological type (Lauren histotype), and ypTMN were all shown in the table. Owing to the form of the data, it was difficult to explore whether there were significant differences (Table 1).

**Table 1.** Basic characteristics of the included literature

	General information			Demographic information of population			Intervention control	Tumor characteristic				Chemotherapy
	First author	Publication year	Country	Sample size	Age	Sex (M/F)	TRG_type	Size (cm)	Location	Histological type (Lauren histotype)	ypTMN	
1	Abborett	2024	Switzerland	74	59 (IQR 51–71)	15/59	Mandard	—	—	Intestinal: 19 Diffuse: 34 Indeterminate: 21	—	MAGIC (EOX, CF)/FLOT/FOLFOX/platine, 5-FU
2	Achilli	2017	Italy	67	67 (range 34–75)	42/25	Becker	—	PROXIMAL third: 19 Middle third: 15 Distal third: 33	Intestinal: 35 Diffuse: 32	1–2: 28 3: 39	ECF/ECX
3	Derieux	2020	France	109	≤60: 47 >60: 62	76/33	Mandard	—	Lower stomach: 52 Upper stomach: 57	Intestinal: 45 Diffuse: 18 Mixed: 46	1–2: 68 3–4: 41	Fluorouracil plus oxaliplatine protocol/cisplatine -based therapy
4	Ikoma	2020	The United States	356	≤65: 215 >65: 141	214/142	—	—	Gastroesophageal junction: 80 Cardia/fundus: 28 Body: 166 Antrum: 66 Total: 16	—	—	5-FU
5	Lombardi	2021	Italy	100	64.81 ± 9.83	64/36	Becker	4.616 ± 2.662	EGJ (Siewert III): 12 Fundus: 20 Body: 30 Antrum: 38	Intestinal: 38 Diffuse: 56 Mixed: 6	1–2: 38 3–4: 62	ECF/FLOT
6	Xie	2021	China/The United States	249	≤65: 159 >60: 90	181/68	—	<5: 117 ≥5: 132	UGEJ: 93 Middle third: 83 Lower third: 57 Diffuse: 16	Intestinal: 64 Diffuse: 185	1–2: 115 3–4: 134	Oxaliplatine-based/nonoxaliplatine-based regimen
7	Xu	2019	China	264	≤60: 159 >60: 105	195/69	Mandard	<5: 203 ≥5: 61	UGEJ: 28 Middle third: 64 Lower third: 172	Intestinal: 157 Diffuse or mixed: 107	1–2: 106 3: 158	SOX/XELOX
8	Gao	2024	China	670	≤55: 180 >55: 490	476/194	Mandard	—	—	Intestinal: 226 Diffuse or mixed: 444	1–2: 169 3–4: 501	FOLFOX/SOX/XELOX
9	Liu	2021	China	413	61(IQR 54–67)	304/109	Mandard	3.0 (1.5–4.0)	UGEJ: 166 Middle third: 51 Lower third: 170 Diffuse: 26	—	—	FOLFOX/SOX/XELOX
10	Tong	2020	China	290	≤65: 221 >65: 69	215/75	Mandard	<5: 115 ≥5: 175	UGEJ: 40 Middle third: 54 Lower third: 172 Diffuse: 24	Intestinal: 143 Diffuse or mixed: 147	1–2: 123 3: 167	Platin-based/taxol-based/triplet regimen
11	Tong	2021	China	141	≤65: 110 >65: 31	97/44	CAP	<5: 50 ≥5: 91	UGEJ: 21 Middle third: 24 Lower third: 81 Diffuse: 15	Intestinal: 71 Diffuse or mixed: 70	1–2: 54 3: 87	FOLFOX/SOX/XELOX

If it cannot be extracted in the original text or is not described in the original text, use —.

EGJ, esophagogastric junction; IQR, interquartile range; TRG, tumor regression grade; UGEJ, upper third and gastroesophageal junction.

### Risk of bias assessment

All 11 articles were scored 7–9, indicating high quality (see Supplementary Table S2, Supplementary Digital Content 1, http://links.lww.com/CTG/B314).

### Summary of findings

#### DFS and TRG

**Figure 2.**
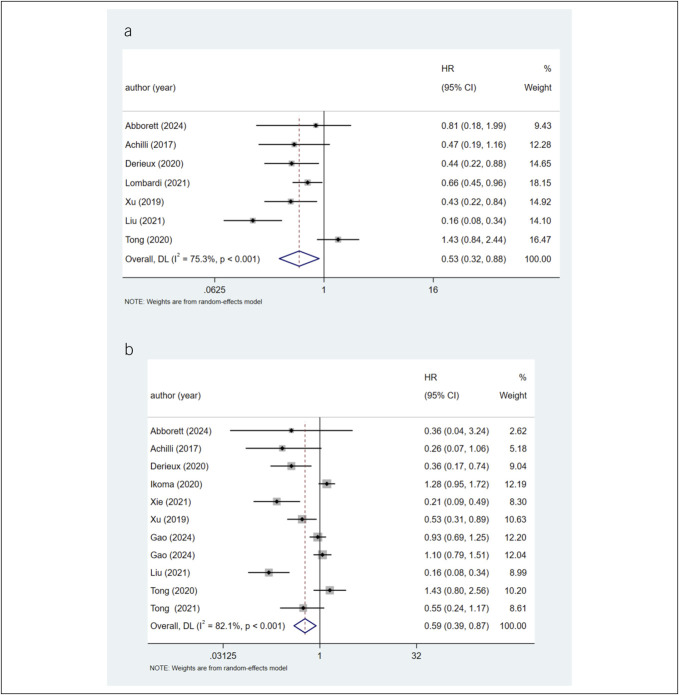
(**a**) Forest plots for the association between TRG and DFS. (**b**) Forest plots for the association between TRG and OS. CI, confidence interval; DFS, disease-free survival; HR, hazard ratio; OS, overall survival; TRG, tumor regression grade.

Seven studies reported data on DFS and TRG. The meta-analysis results indicated that a lower TRG score was associated with prolonged DFS in patients with LAGC receiving neoadjuvant chemotherapy (HR 0.53, 95% CI 0.32–0.88). Given the observed heterogeneity (*I*^2^ = 75.3%), the results suggested that a lower TRG score after treatment in patients with LAGC was linked to prolonged DFS (Figure 2a). Subgroup analyses based on country, sample size, types of TRG, and various grouping methods demonstrated that some results were negative, while others exhibited excessive heterogeneity (see Supplementary Figure S1A–D, Supplementary Digital Content 1, http://links.lww.com/CTG/B314). In addition, there was no significant association of the HR of DFS with sample subtypes (*P* = 0.334, see Supplementary Figure S2, Supplementary Digital Content 1, http://links.lww.com/CTG/B314) and TRG type (*P* = 0.871, see Supplementary Figure S3, Supplementary Digital Content 1, http://links.lww.com/CTG/B314).

#### OS and TRG

Eleven studies investigated the relationship between OS and TRG. A lower TRG score was associated with prolonged OS in patients with LAGC undergoing neoadjuvant chemotherapy (HR 0.59, 95% CI 0.39–0.87). Given the observed heterogeneity (*I*^2^ = 82.1%), the results suggested that a lower TRG score after treatment in patients with LAGC was linked to prolonged OS (Figure 2b). Subgroup analyses based on country, sample size, types of TRG, and various grouping methods demonstrated that some results were negative, while others exhibited excessive heterogeneity (see Supplementary Figure S4A–D, Supplementary Digital Content 1, http://links.lww.com/CTG/B314). In addition, meta-regression showed no significant associations of the HR of OS with sample subtypes (*P* = 0.8, see Supplementary Figure S2, Supplementary Digital Content 1, http://links.lww.com/CTG/B314) and TRG type (*P* = 0.728, see Supplementary Figure S3, Supplementary Digital Content 1, http://links.lww.com/CTG/B314).

#### Sensitivity analysis

**Figure 3.**
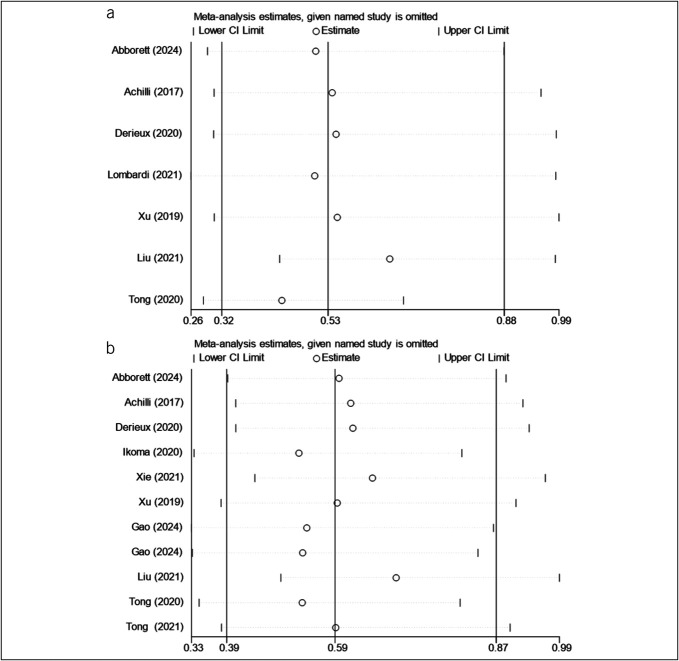
(**a**) Sensitivity analysis of DFS. (**b**) Sensitivity analysis of OS. CI, confidence interval; DFS, disease-free survival; OS, overall survival.

A sensitivity analysis indicated that the effect size remained stable after the sequential removal of each study, suggesting that no single article influenced the results for both DFS (Figure 3a) and OS (Figure 3b), thereby confirming the reliability of the analysis.

#### Publication bias

**Figure 4.**
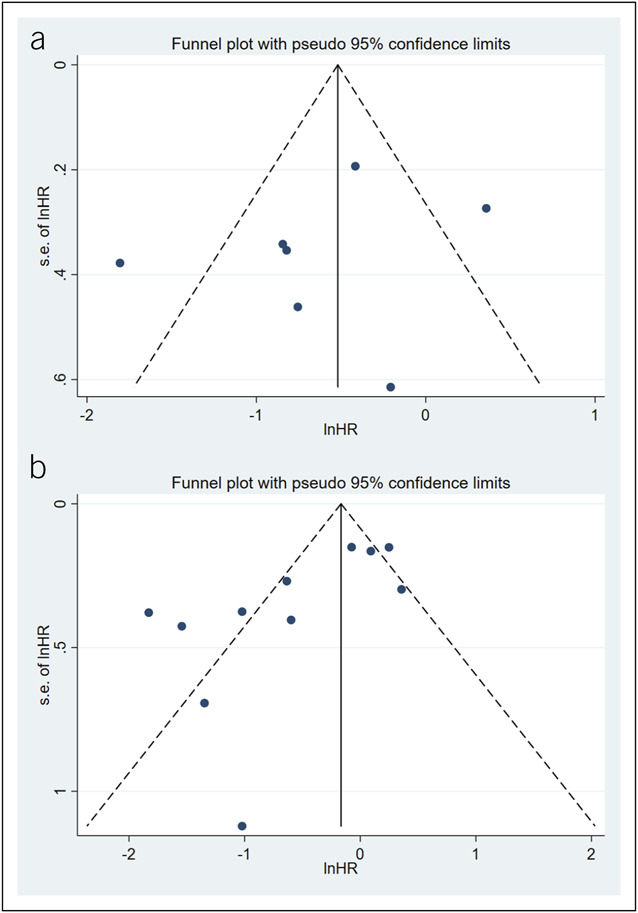
(**a**) Funnel plot for the publication bias for DFS. (**b**) Funnel plot for the publication bias for OS. DFS, disease-free survival; HR, hazard ratio; OS, overall survival.

**Figure 5.**
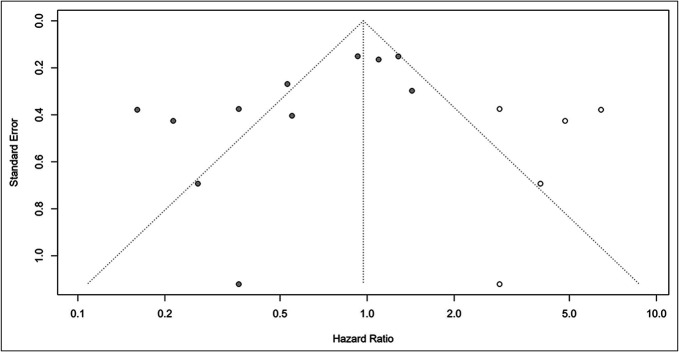
Trim and fill for the publication bias for OS. OS, overall survival.

The symmetrical funnel plot exhibited no significant publication bias in the meta-analysis for DFS (Egger: *P* = 0.458, Figure 4a). However, publication bias was identified in the meta-analysis for OS (Egger: *P* = 0.013, Figure 4b). After editing, 5 new articles were added, and the effect size obtained through the trim-and-fill method was 0.97 (0.55, 1.71, Figure 5).

## DISCUSSION

This study comprehensively analyzed the predictive value of TRG on the prognosis of patients with LAGC receiving neoadjuvant chemotherapy through a systematic review and meta-analysis. The results demonstrated that a lower TRG score was significantly associated with prolonged OS and DFS, and sensitivity analysis further confirmed the robustness of these findings. However, publication bias was detected in the meta-analysis for OS.

From a biological perspective, a low TRG score (indicating marked tumor regression) reflects higher chemosensitivity of tumor cells. Neoadjuvant chemotherapy exerts cytotoxic effects by inducing DNA damage, inhibiting cell proliferation, or disrupting metabolic pathways. Higher chemosensitivity of tumor cells increases apoptosis or necrosis, leading to substantial regression of primary lesions and metastases. For instance, chemotherapeutic agents (e.g., fluorouracil and oxaliplatin) activate caspase-dependent apoptotic pathways or trigger oxidative stress, promoting programmed cell death. Furthermore, reduced residual tumor cell proportions may diminish the survival of cancer stem cells, thereby suppressing the risk of recurrence and metastasis. In addition, alterations in the tumor microenvironment, such as enhanced local inflammatory responses, inhibition of angiogenesis, and increased infiltration of immune cells (e.g., CD8^+^ T cells), may synergistically eliminate residual tumor cells and augment immune surveillance. These mechanisms collectively explain the superior survival outcomes observed in patients with lower TRG scores.

This study has several innovations. First, DFS and OS were included as outcome measures, which enhances the reliability of conclusions because DFS directly reflects tumor-specific control by minimizing interference from non-cancer-related mortality. Second, the use of HR rather than relative risks considers variations in follow-up durations, thus improving methodological rigor. Third, studies from Asia, Europe, and the Americas were included, which addresses the paucity of evidence from Asian populations and strengthens the generalizability of findings. For example, the study by Hayashi et al, which lacked DFS data and had a limited sample size, was complemented by our comprehensive analysis, reinforcing the independent prognostic value of TRG.

This study has several limitations. First, to further explore the source of heterogeneity, information on TRG type, patient baseline characteristics (sample size, age, sex, clinical stage, and comorbidity), tumor characteristics (size, location, and histological type), ypTMN, chemotherapy regimen, tumor biology (HRE2, MSI), and others was collected. If the data were not extracted, it would not be shown in the article. Subgroup analyses were performed based on country, sample size, types of TRG, and various grouping methods. Some results showed significant heterogeneity, likely attributable to differences in patient characteristics and treatment protocols. However, we could not explore it due to significant limitations: heterogeneous reporting (e.g., varying TNM categorization), missing data (lacked tumor biology markers), AND small sample sizes in subgroups precluded meaningful meta-regression. Despite limitations, our analysis provides the largest pooled evaluation of the prognostic utility of TRG. Second, the presence of publication bias in the OS meta-analysis suggests potential overestimation of TRG's impact due to unpublished negative results. Eventually, this deviation was corrected by the trim-and-fill method.

This study provides high-quality evidence supporting TRG as a robust prognostic indicator for patients with LAGC. However, its clinical application should be tailored to individual patient characteristics and complemented by multidimensional biomarkers to optimize prognostic accuracy and therapeutic decision-making.

## CONFLICTS OF INTEREST

**Guarantor of the article:** Lulu Han.

**Specific author contributions:** All authors contributed to the study conception and design. R.W.: writing—original draft preparation. R.W., G.W., and J.C.: writing—review and editing. R.W.: conceptualization and methodology. R.W., S.L., and J.C.: formal analysis and investigation. R.W. and L.H.: funding acquisition, resources, and supervision. All authors commented on previous versions of the manuscript. All authors read and approved the final manuscript.

**Financial support:** The study was supported by Jiangsu Training Program of Innovation and Entrepreneurship for Undergraduates (202310313050Z), National Natural Science Foundation of China (82203172, 82273334, 81871869, and 81400055), The Xuzhou Medical University Excellent Talent Research Start-up Fund (D2021058), The China Postdoctoral Science Foundation (2023M732970).

**Potential competing interests:** None to report.

**Data availability:** The data that support the findings of this study are available from the corresponding author upon reasonable request.

## Supplementary Material
