# Supplementary material for: Predictive Value of Tumor Regression Grading on the Prognosis of Neoadjuvant Chemotherapy for Locally Advanced Gastric Cancer: A Systematic Review and Meta-Analysis
Source: Clin Transl Gastroenterol. 2025 May 14;16(7):e00860. doi: 10.14309/ctg.0000000000000860 (PMC12330353; doi:10.14309/ctg.0000000000000860)
Supplement: Supplementary file 1 [file ct9-16-e00860-s001.pdf]

Supplementary Materials

Table S1 A record of search results

Pubmed		
#	Query	Results
1	Stomach Neoplasms[MeSH Terms]	114732
2	"tumo* of the stomach"[Title/Abstract] OR "tumo* of the gastric"[Title/Abstract] OR "stomach ulcerating tumo*"[Title/Abstract] OR "stomach ulcerated tumo*"[Title/Abstract] OR "stomach tumorigenesis"[Title/Abstract] OR "stomach tumo*"[Title/Abstract] OR "Stomach Neoplas*"[Title/Abstract] OR "stomach mucosa tumo*"[Title/Abstract] OR "stomach malignanc*"[Title/Abstract] OR "stomach cancer*"[Title/Abstract] OR "pyloric cancer"[Title/Abstract] OR "neoplastic stomach"[Title/Abstract] OR "neoplastic gastric"[Title/Abstract] OR "neopla* of the stomach"[Title/Abstract] OR "malignant tumo* of the stomach"[Title/Abstract] OR "malignant neoplasm* of the stomach"[Title/Abstract] OR "malignant gastric tumor"[Title/Abstract] OR "malignant gastric neoplasm"[Title/Abstract] OR "malignanc* of the stomach"[Title/Abstract] OR "gastric tumorigenesis"[Title/Abstract] OR "gastric tumo*"[Title/Abstract] OR "gastric subepithelial tumor"[Title/Abstract] OR "gastric neoplas*"[Title/Abstract] OR "gastric mass* "[Title/Abstract] OR "gastric malignanc*"[Title/Abstract] OR "gastric cardia* cancer"[Title/Abstract] OR "gastric cancer*"[Title/Abstract] OR "gastric body cancer"[Title/Abstract] OR "gastric antrum cancer"[Title/Abstract] OR "gastric antral cancer"[Title/Abstract] OR "cardia cancer"[Title/Abstract] OR "Cancer of the Stomach"[Title/Abstract] OR "cancer of the gastric fundus"[Title/Abstract] OR "cancer of the gastric cardia"[Title/Abstract] OR "cancer of the gastric body"[Title/Abstract] OR "cancer of the gastric antrum"[Title/Abstract] OR "cancer of the cardia"[Title/Abstract] OR "Cancer of Stomach"[Title/Abstract]	105044
3	"tumor regression grade"[Title/Abstract] OR "tumor regression grading"[Title/Abstract] OR "tumour regression grade"[Title/Abstract] OR "tumour regression grading"[Title/Abstract] OR	14295

	"TRG"[Title/Abstract] OR "regression grading"[Title/Abstract] OR "regression grade"[Title/Abstract] OR "tumor regression"[Title/Abstract] OR "tumour regression"[Title/Abstract]	
4	(#1 OR #2) AND #3	446

Embase		
#	Query	Results
1	'stomach cancer'/exp	162733
2	'stomach tumor'/exp	210337
3	'tumor* of the stomach':ti,ab,kw OR 'tumor* of the gastric':ti,ab,kw OR 'ulcerating the stomach*':ti,ab,kw OR 'ulcerating the stomach*':ti, ab,kw OR 'stomach tumorigenesis':ti,ab,kw OR 'stomach tumor*':ti,ab,kw OR 'stomach neoplasm*':ti,ab,kw OR 'stomach mucosal tumor*':ti,ab,kw OR 'stomach malignancy*':ti,ab,kw OR 'stomach cancer*':ti,ab,kw OR pyloric cancer':ti,ab,kw OR 'neoplastic stomach':ti,ab,kw OR 'neoplastic gastric':ti,ab,kw OR 'neopla* of the stomach':ti,ab,kw OR 'malignant tumor* of the stomach':ti,ab,kw OR 'malignant neoplasm* of the stomach':ti,ab,kw OR 'malignant gastric tumor' :ti,ab,kw OR 'malignant gastric neoplasm':ti,ab,kw OR 'malignanc* of the stomach':ti,ab,kw OR 'gastric tumorigenesis':ti,ab,kw OR 'gastric tumor*':ti,ab,kw OR 'gastric subepithelial tumor':ti,ab,kw OR 'gastric neoplasm*': ti,ab,kw OR 'gastric mass*':ti,ab,kw OR 'gastric malignanc*':ti,ab,kw OR 'gastric cardia* cancer':ti,ab,kw OR 'gastric cancer*':ti,ab,kw OR 'gastric body cancer':ti,ab,kw OR 'gastric antrum cancer':ti,ab,kw OR 'gastric antral cancer':ti,ab,kw OR 'cardia cancer':ti,ab,kw OR 'cancer of the stomach':ti,ab,kw OR 'cancer of the gastric fundus':ti,ab,kw OR 'cancer of the gastric cardia':ti,ab,kw OR 'cancer of the gastric body':ti,ab,kw OR 'cancer of the gastric antrum':ti,ab,kw OR 'cancer of the heart':ti,ab,kw OR 'cancer of the stomach':ti,ab,kw	140583
4	'tumor regression grade':ti,ab,kw OR 'tumor regression grading':ti,ab,kw OR 'tumour regression grade':ti,ab,kw OR 'tumour regression grading':ti,ab,kw OR 'trg':ti,ab,kw OR 'regression grading':ti,ab,kw OR 'regression grade':ti,ab,kw OR 'tumor	23417

	regression':ti,ab,kw OR 'tumour regression':ti,ab,kw	
5	(#1 OR #2 OR #3) AND #4	810

Cochrane Library		
#	Query	Results
1	MeSH descriptor: [Stomach Neoplasms] explode all trees	4077
2	('tumo* of the stomach' OR 'tumo* of the gastric' OR 'stomach ulcerating tumo*' OR 'stomach ulcerated tumo*' OR 'stomach tumorigenesis' OR 'stomach tumo*' OR 'Stomach Neoplas*' OR 'stomach mucosa tumo*' OR 'stomach malignanc*' OR 'stomach cancer*' OR 'pyloric cancer' OR 'neoplastic stomach' OR 'neoplastic gastric' OR 'neopla* of the stomach' OR 'malignant tumo* of the stomach' OR 'malignant neoplasm* of the stomach' OR 'malignant gastric tumor' OR 'malignant gastric neoplasm' OR 'malignanc* of the stomach' OR 'gastric tumorigenesis' OR 'gastric tumo*' OR 'gastric subepithelial tumor' OR 'gastric neoplas*' OR 'gastric mass* ' OR 'gastric malignanc*' OR 'gastric cardia* cancer' OR 'gastric cancer*' OR 'gastric body cancer' OR 'gastric antrum cancer' OR 'gastric antral cancer' OR 'cardia cancer' OR 'Cancer of the Stomach' OR 'cancer of the gastric fundus' OR 'cancer of the gastric cardia' OR 'cancer of the gastric body' OR 'cancer of the gastric antrum' OR 'cancer of the cardia' OR 'Cancer of Stomach'):ti,ab,kw	15391
3	('tumor regression grade' OR 'tumor regression grading' OR 'tumour regression grade' OR 'tumour regression grading' OR 'TRG' OR 'regression grading' OR 'regression grade' OR 'tumor regression' OR 'tumour regression'):ti,ab,kw	9422
4	(#1 OR #2) AND #3	334

WOS		
#	Query	Results
1	TS=("tumo* of the stomach" OR "tumo* of the gastric" OR "stomach	120187

	ulcerating tumo*" OR "stomach ulcerated tumo*" OR "stomach tumorigenesis" OR "stomach tumo*" OR "Stomach Neoplas*" OR "stomach mucosa tumo*" OR "stomach malignanc*" OR "stomach cancer*" OR "pyloric cancer" OR "neoplastic stomach" OR "neoplastic gastric" OR "neopla* of the stomach" OR "malignant tumo* of the stomach" OR "malignant neoplasm* of the stomach" OR "malignant gastric tumor" OR "malignant gastric neoplasm" OR "malignanc* of the stomach" OR "gastric tumorigenesis" OR "gastric tumo*" OR "gastric subepithelial tumor" OR "gastric neoplas*" OR "gastric mass* " OR "gastric malignanc*" OR "gastric cardia* cancer" OR "gastric cancer*" OR "gastric body cancer" OR "gastric antrum cancer" OR "gastric antral cancer" OR "cardia cancer" OR "Cancer of the Stomach" OR "cancer of the gastric fundus" OR "cancer of the gastric cardia" OR "cancer of the gastric body" OR "cancer of the gastric antrum" OR "cancer of the cardia" OR "Cancer of Stomach")	
2	TS=("tumor regression grade" OR "tumor regression grading" OR "tumour regression grade" OR "tumour regression grading" OR "TRG" OR "regression grading" OR "regression grade" OR "tumor regression" OR "tumour regression")	15677
3	#1 AND #2	449

CNKI		
#	Query	Results
Full text	Carcinoma of stomach + "gastric cancer" + "gastric carcinoma" + "stomach cancer" + stomach neoplasm （ synonym expanded, exact ）	
Full text	Tumor regression grade + Tumor regression grade (synonym expanded, exact)	

VIP		
#	Query	Results

Title or keywords, vague	Gastric cancer+gastric carcinoma+stomach cancer+gastric tumors (year of database establishment- 2024; all journals; all)
Any field, fuzzy	Tumor regression grade + tumor regression grade (year of database establishment - 2024; all journals; all)

WF		
#	Query	Results
All, Blur	Gastric cancer OR gastric cancer OR gastric carcinoma OR stomach cancer OR gastric neoplasms (journals, conferences, papers; synonym expansion, subject heading expansion; no limit - to date)	
All, Blur	Tumor regression grade OR Tumor regression grade (journal, conference, paper; synonym expansion, subject heading expansion; no limit - to date)	

Sino Med		
#	Query	Results
All fields	Gastric cancer OR gastric cancer OR gastric carcinoma OR stomach cancer OR stomach tumor	
All fields	Tumor regression grade OR Tumor regression grade	

Table S2 Newcastle-Ottawa Scale

NO	exposed group representativeness	non-exposed group selection	exposure determination	outcome at start	comparability consideration 1	comparability consideration 2	outcome assessment adequacy	sufficient follow-up duration	adequate follow-up	score
1	1	1	1	1	1	1	1	1	1	9
2	1	1	1	1	1	0	1	0	1	7
3	1	0	1	0	1	1	1	1	1	7
4	1	1	1	1	1	NR	1	1	1	8
5	1	1	1	1	1	0	1	0	1	7
6	1	1	1	1	1	1	1	1	1	9
7	1	1	1	1	1	1	1	NR	1	8
8	1	1	1	1	0	1	1	1	1	8
9	1	1	1	NR	1	1	1	1	1	8
10	1	1	1	1	1	1	1	0	0	7
11	1	1	1	1	1	1	1	1	1	9

Supplementary Figures

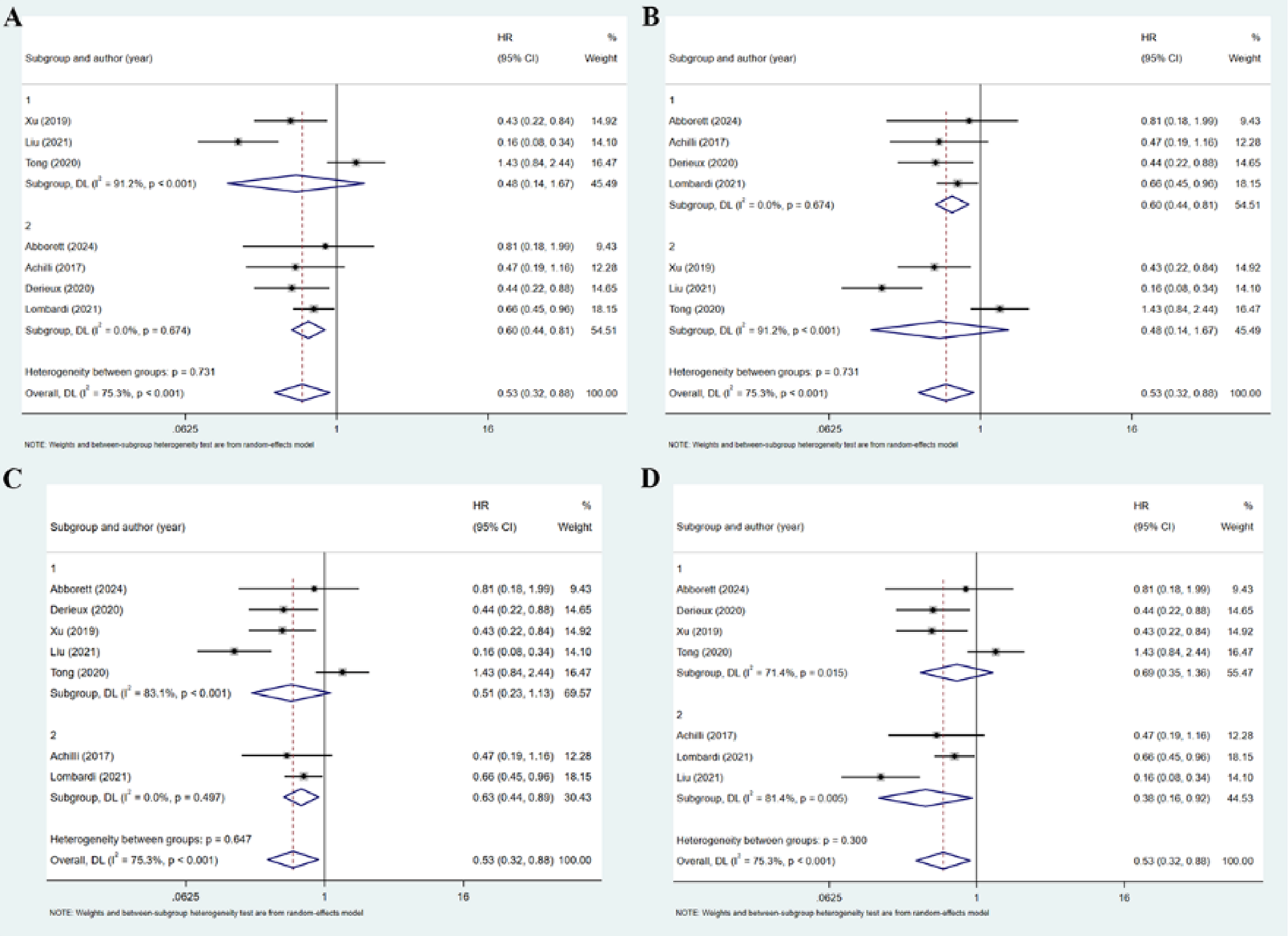

Figure S1 (A) Forest plots for the association between TRG and OS in country; (B) Forest plots for the association between TRG and OS in sample; (C) Forest plots for the association between TRG and OS in type of TRG; (D) Forest plots for the association between TRG and OS in group by

A

Meta-regression	Number of obs	=	11
REML estimate of between-study variance	tau2	=	.4589
% residual variation due to heterogeneity	I-squared_res	=	80.61%
Proportion of between-study variance explained	Adj R-squared	=	2.41%
With Knapp-Hartung modification			

lnHR	Coef.	Std. Err.	t	P>|t|	[95% Conf. Interval]
sample	.5394947	.5284577	1.02	0.334	-.6559596 1.734949
_cons	-1.493131	.942743	-1.58	0.148	-3.625764 .6395016

B

Meta-regression	Number of obs	=	7
REML estimate of between-study variance	tau2	=	.4365
% residual variation due to heterogeneity	I-squared_res	=	79.42%
Proportion of between-study variance explained	Adj R-squared	=	-25.87%
With Knapp-Hartung modification			

lnHR	Coef.	Std. Err.	t	P>|t|	[95% Conf. Interval]
sample	-.154168	.5764603	-0.27	0.800	-1.636006 1.32767
_cons	-.4097938	.8847396	-0.46	0.663	-2.684089 1.864502

Figure S2 (A) meta-regression for sample of DFS; (B) meta-regression for sample of OS

A

Meta-regression Number of obs = 7
REML estimate of between-study variance tau2 = .4372
% residual variation due to heterogeneity I-squared_res = 79.29%
Proportion of between-study variance explained Adj R-squared = -26.09%
With Knapp-Hartung modification

lnHR	Coef.	Std. Err.	t	P>|t|	[95% Conf. Interval]
trg_type	.1070384	.6262237	0.17	0.871	-1.502721 1.716798
_cons	-.7728388	.8634732	-0.90	0.412	-2.992467 1.44679

B

Meta-regression Number of obs = 9
REML estimate of between-study variance tau2 = .4661
% residual variation due to heterogeneity I-squared_res = 80.27%
Proportion of between-study variance explained Adj R-squared = -14.98%
With Knapp-Hartung modification

lnHR	Coef.	Std. Err.	t	P>|t|	[95% Conf. Interval]
trg_type	-.1461893	.4031349	-0.36	0.728	-1.099452 .8070734
_cons	-.3826685	.586222	-0.65	0.535	-1.768863 1.003526

Figure S3 (A) meta-regression for trg_type of DFS; (B) meta-regression for trg_type of OS

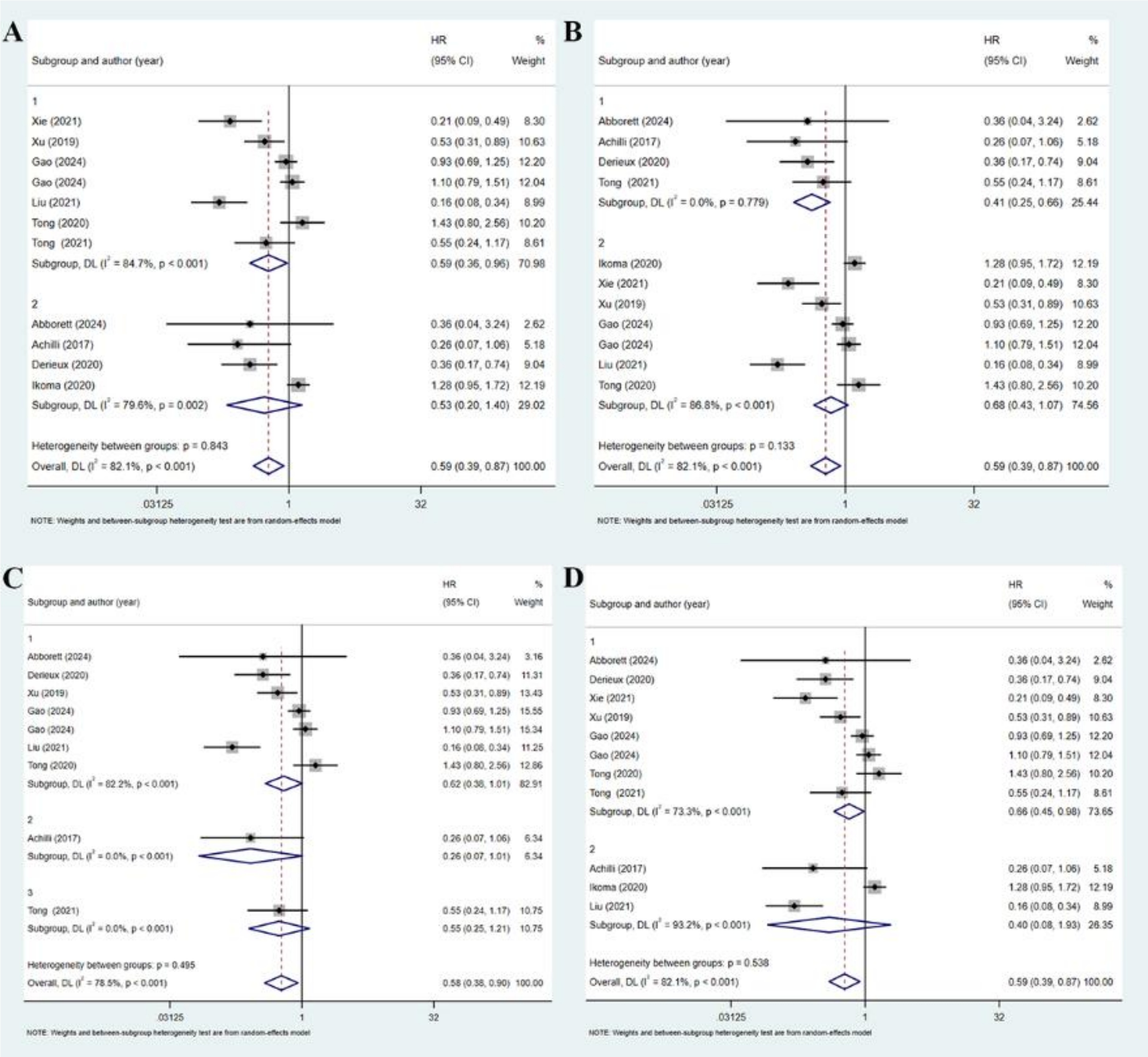

Figure S4 Forest plots for the association between TRG and DFS in country; (B) Forest plots for the association between TRG and DFS in sample; (C) Forest plots for the association between TRG and DFS in type of TRG; (D)Forest plots for the association between TRG and DFS in group by
